# The Cost and Educational Experience of Treating Supracondylar Humerus Fractures: A Pilot Analysis on Standardizing Surgical Care

**DOI:** 10.5435/JAAOSGlobal-D-20-00063

**Published:** 2020-06-01

**Authors:** Alvin W. Su, Mark C. Lee

**Affiliations:** From the Department of Orthopedic Surgery (Dr. Su, Dr. Lee), Connecticut Children's Medical Center, University of Connecticut, Hartford, CT, and the Department of Orthopedic Surgery (Dr. Su), Nemours Alfred I. duPont Hospital for Children, Wilmington, DE.

## Abstract

Closed reduction and percutaneous pinning (CRPP) for supracondylar humeral fractures (SCHF) comprised considerable surgical volume in pediatric orthopaedics. Limited reports are available on how standardization of the surgical care affects the cost and trainee's learning experience.

**Methods:**

Cost analysis was performed by chart review with the billing department in a university teaching hospital. The association of cost with perioperative variables was determined by univariate and multivariable analyses. The educational experience was acquired by questionnaires completed by seven attending surgeons and 22 orthopaedic trainees.

**Results:**

Fifty-one patients were included, revealing the hospital charge of $6,345 per CRPP case. Most of the cost comprised OR time (67%) and anesthesia time (13%). The attending surgeon and fracture type were independently associated with anesthesia time. Standardization of care was perceived for better learning experience and cost saving.

**Conclusion:**

Efforts in the standardization of SCHF surgical care can improve cost saving and trainees' learning experience.

Supracondylar humeral fractures (SCHF) are the most common type of elbow fractures in children. The average total hospital charge to treat SCHF has been recently reported as $17,865 with surgical intervention and $2,965 if managed in the emergency department (ED) only, based on the Nationwide Emergency Department (ED) Sample Database.^1^ The transition in the healthcare system to value-based care, together with the ongoing implementation of fixed reimbursement in lieu of fee-for-service models, have motivated cost awareness in most orthopaedic practices.

SCHF comprise considerable surgical volume in pediatric orthopaedics. When surgery is indicated, most of these cases are managed with closed reduction and percutaneous pinning (CRPP), which is an important part of orthopaedic trainees' education and one of the few surgical competencies in pediatric orthopaedics evaluated by the ACGME (Accreditation Council for Graduate Medical Education). There have been continued efforts on improving the trainees' experience and clinical competency, such as integrating video^2,3^ and internet-smartphone^3^ technologies, using more specialty-oriented assessment tools^3,4^ and participating in quality improvement processes.^5^ On the other hand, sufficient hands-on repetition with mental rehearsals and an active learning attitude can tremendously benefit the development toward surgical competency.^6^

Standardizing surgical procedures improves efficiency, quality of care,^7,8^ and may potentially smoothen the learning curve for trainees. Understanding the source of the cost can facilitate cost saving. Besides promoting cost-effectiveness from a medical-economic point of view, it is also imperative not to jeopardize but to improve the trainees' educational experience in an academic institution. To our knowledge, there have been limited reports focusing on the cost analysis of SCHF and its influence on orthopaedic education. We hypothesized that standardizing the perioperative procedures saves cost and improves trainees' learning experience. The aims of the study were to assess the following: (1) the cost of surgically treating SCHF and (2) the effect of standardizing the perioperative procedures on trainees' educational experience and potential cost saving.

## Methods

The present study consisted of the following two parts: (1) cost analysis and (2) orthopaedic trainees' educational experience. The investigation was conducted in a tertiary referral children's hospital, which is also the teaching hospital for a university orthopaedic surgery residency program.

### Cost Analysis

The studied cohort was identified by a retrospective search through the billing department in our institution, for a consecutive three-month time frame, using the CPT (Current Procedural Terminology) code 24538: “Percutaneous skeletal fixation of supracondylar or transcondylar humeral fracture, with or without intercondylar extension.”

After identifying the cases, each patient's demographic variables and surgical variables were documented on a chart review. The demographic variables included the age and Body Mass Index (BMI, kg/m^2^). The surgical variables included the fracture type (Gartland type II vs. type III vs. flexion type), the postgraduate year (PGY) level of the trainee on the case (from PGY1 to PGY6, then further categorized as—“junior: PGY1-3” vs. “senior: PGY4 or above”), the number of K-wires used for fracture fixation, the anesthesia time, the OR time, and the attending surgeon of the case (surgeon #1 to #6).

The cost data were provided by the billing department in the form of hospital charges. The cost items included those incurred within the time frame from the moment the operating suite started to prepare for the case on the patient's arrival to the room until the patient was escorted out of the operating suite and the room was cleaned up. The cost items were then categorized into the followings: operating room (OR) time, anesthesia time, radiology for intraoperative fluoroscopy, IV therapy, medications, implants (K-wires), and miscellaneous medical supplies other than implants (including the cast or splint, dressings, etc.).

### Orthopaedic Trainees' Educational Experience

Two distinct survey questionnaires were completed by (1) seven pediatric orthopaedic attending surgeons, who estimated the OR-time saved when teaching the residents and the marginal cost saving that would motivate the change of personal routines, including perioperative logistics, reduction and casting techniques, anesthesia, and radiology preferences and (2) 22 orthopaedic residents assessing how standardization affects case numbers and time required to reach competency of independence (Appendix 1).

### Statistical Analyses

Univariate analyses were first performed to assess the effect of each demographic and surgical variable on the anesthesia time, which best reflects the operative procedure from positioning the patient to finishing the cast or splint. The effect of the patient's age, BMI, and the number of K-wires used for fracture fixation was assessed by the linear correlation test. The effect of who the attending surgeon was, the fracture type, and the PGY level of the trainee was assessed by one-way analysis of variance or unpaired two-tailed Student *t*-test as appropriate.

The multivariable regression model was then constructed after identifying potential confounding variables. All variables that showed the trend toward association with the change in the anesthesia time based on univariate analyses were considered for inclusion in the multivariable analysis to assess the possible independent association between the studied variables with the anesthesia time. After ascertainment of such an association, odds ratios (OR) and 95% confidence intervals were estimated for each possible association.

Descriptive statistics were used to assess the distribution of the costs and the survey questionnaire data. To further analyze the survey data, the association between the PGY level, SCHF CRPP case log numbers, and the trainee's confidence in performing CRPP for SCHF as the primary surgeon using the perceived anesthesia time as a surrogate was assessed by the chi-square test or the Fisher exact test (when any cell count <5 in a 2 × 2 contingency table) as appropriate.

Significance was set at α = 0.05.

## Results

There were a total of 51 patients (51 cases) identified and included in the present study. The mean age was 5.9 years (70.5 months). All of these patients achieved routine osseous union without documented complications or return-to-OR for treating of the same fracture. The mean BMI was 16.5 kg/m^2^. The mean anesthesia and OR times were 61.2 and 182.7 minutes, respectively. There were 14 junior (PGY1-3) and eight senior (PGY 4 & above) trainees who participated in the survey questionnaire.

**Figure 1:**
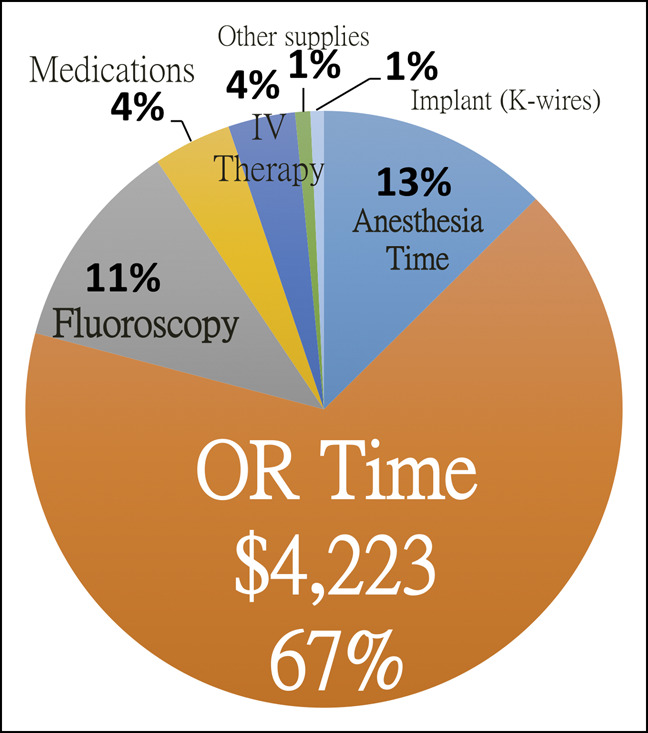
Graph demonstrating the distribution of the cost of surgically treating SCHF with CRPP, *not* including the cost of ED visit ($2,151 and above per visit), if any, and the cost throughout the time in the postanesthesia care unit (PACU, $24 per minute). CRPP = closed reduction and percutaneous pinning; SCHF = supracondylar humeral fractures.

The average cost for the CRPP of one SCHF case was $6,345 of hospital charge. The OR time comprised 67% of the cost ($4,223 in total, $23/minute), followed by that of the anesthesia time of 13% ($823 in total, $13/minute) and the intraoperative fluoroscopy of 11% ($819 in total). Of note, the fiberglass materials used for casts comprised less than 1% of the total cost, with the charge of $4 to 6 per roll and in rare exceptions of $31 per roll for certain special color-pattern designs. The cost of ED visit (ED, from $2,151 and above per visit) and the time spent in the post anesthesia care unit ($24 per minute) were not included in our analysis. The cost of hospital admission, if any, was not included either (Figure 1).

On univariate analyses, increased patient's age (*R*^2^ = 0.10, *P* = 0.024) and the number of K-wires used for fracture fixation (R^2^ = 0.09, *P* = 0.036) were correlated with the anesthesia time. BMI (*R*^2^ = 0.05, *P* = 0.099) trended toward weak correlation with the anesthesia time. The attending surgeon of the case (*P* < 0.001) and the SCHF type (*P* = 0.001) were associated with the anesthesia time. The trainee's PGY level did not show statistically significant association (*P* = 0.138) with the anesthesia time (Table 1).

**Table 1:** Demographics and Clinical Characteristics of the Patients Who Underwent CRPP of SCHF on (A) Univariate Analyses and (B) Linear Correlation Test

(A) Variable	Anesthesia Time (Mean ± SD, minutes)	*P* Value
Attending surgeon (#1-#7)		0.0001
#1	45.8 ± 7.1	
#2	64.7 ± 16.0	
#3	62.3 ± 8.6	
#4	72.2 ± 21.7	
#5	78.3 ± 17.6	
#6	47.2 ± 7.3	
#7	63.0 ± 15.6	
SCHF type (flexion vs. II vs. III)		0.0012
Flexion type	95.0 ± 13.1	
Type II	56.8 ± 13.6	
Type III	61.2 ± 17.8	
PGY level of the trainee		0.14
Junior (PGY1-3)	56.4 ± 13.8	
Senior (PGY4 & above)	64.1 ± 19.4	

(B) Variable	All Patients	*R*^2^	*P* Value
Anesthesia time (min)	61.2 ± 17.8	n/a	n/a
No. of K-wires used for fixation	2.5 ± 0.5 # of K-wires	0.09	0.04
BMI (kg/m^2^)	16.5 ± 3.1	0.10	0.02
Age (mo)	70.5 ± 24.5	0.05	0.33

BMI, body mass index; CRPP = closed reduction and percutaneous pinning; PGY: post-graduate year; SCHF, supracondylar humerus fracture

On multivariable regression analysis, both the attending surgeon of the case and the SCHF type were independently associated with the anesthesia time. One of the seven attending surgeons was associated with decreased anesthesia time (*P* = 0.023), whereas another attending surgeon trended toward decreased anesthesia time (*P* = 0.066). The flexion fracture type was associated with increased anesthesia time (*P* = 0.023) (Table 2).

**Table 2:** Multivariable Regression Analysis on the Independent Association Between the Studied Variables and Anesthesia Time

Variable	Regression Coefficient (95% CI)	*P* Value
Attending surgeon		
#1	**−25.40 (−45.31 to −5.48)**	**0.01**
#2	−4.44 (−25.42 to 16.53)	0.67
#3	−5.56 (−25.72 to 14.59)	0.58
#4	6.66 (−13.61 to 26.92)	0.51
#5	−0.53 (−23.79 to 22.73)	0.96
#6	−20.49 (−42.42 to 1.44)	0.07
#7	0	Ref
SCHF type		
Flexion type	**23.87 (3.44 to 44.30)**	**0.02**
Type II	−5.59 (−16.85 to 5.66)	0.32
Type III	0	Ref
PGY level of the trainee		
Junior (PGY1-3)	−6.61 (−15.11 to 1.89)	0.124
Senior (PGY4 & above)	0	Ref
No. of K-wires used for fixation	7.47 (−2.66 to 17.60)	0.14
BMI (kg/m^2^)	0.021 (−1.42 to 1.47)	0.98
Age (mo)	0.08 (−0.09 to 0.25)	0.33

BMI = body mass index; CI = confidence interval; PGY = post-graduate year; Ref = reference group; SCHF = supracondylar humerus fracture. Bold face indicates significant difference with P<0.05.

Using the trainees' projected surgical time as a surrogate, higher number of cases logged was associated with higher confidence level as the primary surgeon performing CRPP (Table 3), whereas the PGY level did not show statistical significance regarding their confidence level (Table 4). However, five of the 14 junior trainees did not feel capable of being the primary surgeon, for which all of the eight senior trainees felt comfortable with.

**Table 3:** Association of Higher Logged Case Numbers With Higher Confidence Level of Performing CRPP for SCHF Using the Projected Surgical Time as a Surrogate

Chi-Square Test	The “Anesthesia Time” I Need From Positioning Until Finishing the Cast			*P* Value
	<30 min	<60 min	I Can't Do It	
Case log #				
1-5 cases	1	3	5	0.006
≥6 cases	7	6	0	

CRPP = closed reduction and percutaneous pinning; SCHF = supracondylar humeral fractures

**Table 4:** Nonassociation of PGY Level With the Confidence Level of Performing CRPP for SCHF

Chi-Square Test	The “Anesthesia Time” I Need From Positioning Until Finishing the Cast			*P* Value
	<30 min	<60 min	I Cannot Do It	
PGY level				
PGY 1-3	4	5	5	0.153
PGY 4-6	4	4	0	

CRPP = closed reduction and percutaneous pinning; PGY = post-graduate year; SCHF = supracondylar humeral fractures

Almost all trainees (21 of 22) felt that it would benefit their training experience in treating SCHF, if every attending surgeon manages the case the same way (Table 5A).

**Table 5:** (A) The Orthopaedic Trainees' Perception of Standardizing the Surgical Care Regarding Their Learning Experience, (B) Expected Time Added to CRPP of SCHF Based on the Attending Surgeons' Experience, and (C) Incentives in Cost Saving and Surgical Time to Motivate Change in an Attending Surgeon's Routines

(A) Answer Choices	Responses (%, n)	
Do you think it would benefit your training if every attending treated supracondylar humerus fractures surgically the same way?		
Yes	95.45%	21
No	4.55%	1

(B) Answer Choices	Responses (%, n)	
How much extra time does a junior resident add to surgical treatment for a supracondylar humerus fracture if he/she has never operated on this fracture before? (as opposed to doing it all on your own)		
∼0 minute (as quickly as I do it myself)	14.29%	1
∼5 minutes	0.00%	0
∼10 minutes	42.86%	3
∼15 minutes or more	42.86%	3
How much extra time does a junior resident add to surgical treatment for a supracondylar humerus fracture if he/she has been trained by you MORE THAN THREE times before?		
∼0 minute (as quickly as I do it myself)	57.14%	4
∼5 minutes	28.57%	2
∼10 minutes	14.29%	1
∼15 minutes or more	0.00%	0
How much extra time does a senior resident or fellow add to surgical treatment for a supracondylar humerus fracture if he/she has been trained by another attending? (as opposed to doing it all on your own)		
∼0 minute (as quickly as I do it myself)	57.14%	4
∼5 minutes	28.57%	2
∼10 minutes	14.29%	1
∼15 minutes or more	0.00%	0

(C) Answer Choices	Responses (%, n)	
What % of cost savings would prompt you to change the way taking care of supracondylar humerus fracture patients?		
10%	0.00%	0
30%	71.43%	5
More than 50%	14.29%	1
Prefer not to change, regardless of the cost savings.	14.29%	1
What amount of time saved teaching trainees in the OR would prompt you to change the way fixing a supracondylar humerus fracture?		
∼5 minutes	0.00%	0
∼10 minutes	14.29%	1
∼15 minutes	42.86%	3
∼30 minutes	42.86%	3
60 minutes or more	0.00%	0
Which component of the supracondylar fracture patient care would you be willing to change (standardized)? (please check all that apply)		
Settings in the OR (positioning, C-arm, draping, antibiotics, and anesthesia method)	57.14%	4
Fracture reduction and fixation technique	28.57%	2
Casting techniques and dressing materials (Xeroform, 4 × 4, color/white fiberglass/plasters…)	71.43%	5
Pain control (NSAIDs, narcotics)	71.43%	5
Peri-OP logistics (pre-OP admission vs. day surgery, etc., pre-OP splint, post-OP follow-up visit frequency, and management…)	100.00%	7
Prefer NOT to change anything	0.00%	0
Which one component of supracondylar fracture patient care is least likely to change in your hands, regardless of potential savings in time and cost?		
Settings in the OR (positioning, C-arm, draping, antibiotics, and anesthesia method)	14.29%	1
Fracture reduction and fixation technique	57.14%	4
Casting techniques and dressing materials (Xeroform, 4 × 4, color/white fiberglass/plasters…)	0.00%	0
Pain control (NSAIDs, narcotics)	0.00%	0
Peri-OP logistics (pre-OP admission vs. day surgery, etc., pre-OP splint, post-OP follow-up visit frequency, and management…)	0.00%	0
I am open to change in anything for cost and/or time savings	28.57%	2

CRPP = closed reduction and percutaneous pinning; OP = operative, OR = operating room, SCHF = supracondylar humeral fractures

The additional OR time spent on teaching the trainees was perceived to decrease because the trainee was repeatedly exposed to the same procedural routine. Six of the seven attending surgeons reported that to teach a junior resident to perform CRPP would add 10 minutes or more of the procedure time if the resident had never done it before. If the junior resident has been trained on SCHF cases with the same attending surgeon before for three times or more, six of the seven attending surgeons reported no addition (n = 4) or additional 5 minutes (n = 2) or the procedure time. Such trend was less obvious with senior trainees (Table 5B).

The cost saving of 30% or more or the OR time saving of 15 minutes or more would likely motivate most attending surgeons to change their routines. All seven attending surgeons were open to the idea of changing their perioperative logistics routines, whereas more than half of the surgeons preferred not to change their fracture reduction and fixation technique (Table 5C).

## Discussion

The present study showed that standardizing the surgical routine of treating SCHF can potentially save costs mostly by saving OR time and benefit the learning curve of the orthopaedic trainees. Given sufficient incentives in cost savings, it seems that most surgeons would be willing to look into certain routine modifications. Repetition remains the cornerstone of reaching surgical competency, based on the trainees' experience.

Our study has several limitations. First, the analyses included only limited number of cases and trainees by sampling of a given period of time in a year. Different time frames may reveal variability in pateint and trainee demographics. Limited samples may have jeopardized the statistical power especially for the logistic regression model. Nevertheless, we think that sampling of three months in a year could provide decent perspectives in cost distribution. However, it must be recognized that the hospital billing records serve as an estimation and may not reflect the multiple facets of perioperative costs. Second, the “time” documented for each case may be influenced by factors other than the surgeon and the trainee, such as equipment setups and patients' response to anesthesia. Third, the questionnaires represented subjective experiences and may not reflect the real-life scenarios, such as the actual time saving by repetition of same surgical routines. Last but not the least, our study did not measure the long-term patient outcomes, although all patients achieved osseous union without complications.

With the increasing awareness of cost-effectiveness in our healthcare system, the “effectiveness,” encompassing the patient's safety and quality of the care, in our belief, should not be compromised for the sole purpose of minimizing the cost. Efforts have been ongoing to improve quality, and at the same time, save cost managing pediatric fractures, from various approaches such as optimizing pain control protocols.^9,10^ Similarly, if one set of surgical routines can save costs and achieve better effectiveness on training experience without compromising patient outcomes, standardizing the surgical care would be well worth the effort.

Standardizing care of pediatric fractures involve active participation of the clinical care team, especially the treating clinicians. Implementation of evidence-based protocols for quality improvement of surgical care can better motivate the surgeons to comply.^11^ As surgeons, it seems that we prefer not to change our own particular techniques of fracture reduction and implant fixation, namely “what works in my hands.” On the other hand, our results implied that cost saving, including the time saved teaching the trainees, could be quite appealing to prompt change in surgical routines. In addition, we feel that as surgeons, we are always looking for “better ways to do things.”

Repetition of the same steps in the same type of procedure has always been an essential component of a surgeon's maturation. In addition, standardizing the surgical routines can potentially alleviate the OR staff's burden and anxiety, benefiting from the simplified surgeons' preference card^11^ and a more predictable, smoother perioperative flow, regardless of who the staff would be for the case. It is reasonable to assume that standardizing such surgical routines is typically welcomed by the OR staff, as long as the surgeons can reach consensus.

We sought to contribute to the ongoing endeavors among colleagues in improving the cost-effectiveness of surgical care of SCHF. Our analyses were based on a single teaching institution, and we hope to share these pilot experiences as we keep cultivating a standardized protocol that would benefit the surgeons, trainees, staff, and thus the institution as a whole. Moreover, collaboration with the anesthesiologists to integrate their workflow into standardization may further substantiate time and cost saving. Prospective data with more accurate quantification of the variables such as time, cost, and subjective and objective patient outcomes could provide more solid evidence, thus leading to practice advice on cost-effective improvement. Patient safety and quality of care remain our first and foremost concern as we continue our research to improve cost-effectiveness.

## Appendix 1 Questionnaires Completed by (A) Seven Pediatric Orthopaedic Attending Surgeons and (B) 22 Pediatric Orthopaedic Trainees

**Table:**

(A) Answer Choices	Responses (%, n)	
Q1. How much extra time does a junior resident add to surgical treatment for a supracondylar humerus fracture if he/she has never operated on this fracture before? (as opposed to doing it all on your own)		
∼0 minute (as quickly as I do it myself)	14.29%	1
∼5 minutes	0.00%	0
∼10 minutes	42.86%	3
∼15 minutes or more	42.86%	3
Q2. How much extra time does a junior resident add to surgical treatment for a supracondylar humerus fracture if he/she has been trained by another attending? (as opposed to doing it all on your own)		
∼0 minute (as quickly as I do it myself)	14.29%	1
∼5 minutes	57.14%	4
∼10 minutes	14.29%	1
15 minutes or more	14.29%	1
Q3. How much extra time does a junior resident add to surgical treatment for a supracondylar humerus fracture if he/she has been trained by you ONE time before?		
∼0 minute (as quickly as I do it myself)	14.29%	1
∼5 minutes	71.43%	5
∼10 minutes	0.00%	0
∼15 minutes or more	14.29%	1
Q4. How much extra time does a junior resident add to surgical treatment for a supracondylar humerus fracture if he/she has been trained by you MORE THAN THREE times before?		
∼0 minute (as quickly as I do it myself)	57.14%	4
∼5 minutes	28.57%	2
∼10 minutes	14.29%	1
∼15 minutes or more	0.00%	0
Q5. How much extra time does a senior resident or fellow add to surgical treatment for a supracondylar humerus fracture if he/she has been trained by another attending? (as opposed to doing it all on your own)		
∼0 minute (as quickly as I do it myself)	57.14%	4
∼5 minutes	28.57%	2
∼10 minutes	14.29%	1
∼15 minutes or more	0.00%	0
Q6. How much extra time does a senior resident or fellow add to surgical treatment for a supracondylar humerus fracture if he/she has been trained by you ONE time before?		
∼0 minute (as quickly as I do it all by myself)	57.14%	4
∼5 minutes	28.57%	2
∼10 minutes	14.29%	1
∼15 minutes or more	0.00%	0
Q7. How much extra time does a senior resident or fellow add to surgical treatment for a supracondylar humerus fracture if he/she has been trained by you MORE THAN THREE times before?		
∼0 minutes (as quickly as I do it all by myself)	85.71%	6
∼5 minutes	14.29%	1
∼10 minutes	0.00%	0
∼15 minutes or more	0.00%	0
Q8. What % of cost savings would prompt you to change the way taking care of supracondylar humerus fracture patients?		
10%	0.00%	0
30%	71.43%	5
More than 50%	14.29%	1
Prefer not to change, regardless of the cost savings.	14.29%	1
Q9. What amount of time saved teaching trainees in the OR would prompt you to change the way fixing a supracondylar humerus fracture?		
∼5 minutes	0.00%	0
∼10 minutes	14.29%	1
∼15 minutes	42.86%	3
∼30 minutes	42.86%	3
60 minutes or more	0.00%	0
Q10. Which component of the supracondylar fracture patient care would you be willing to change (standardized)? (please check all that apply)		
Settings in the OR (positioning, C-arm, draping, antibiotics, anesthesia method)	57.14%	4
Fracture reduction and fixation technique	28.57%	2
Casting techniques and dressing materials (Xeroform, 4 × 4, color/white fiberglass/plasters…)	71.43%	5
Pain control (NSAIDs, narcotics)	71.43%	5
Peri-OP logistics (pre-OP admission vs. day surgery, etc., pre-OP splint, post-OP follow-up visit frequency, and management…)	100.00%	7
Prefer NOT to change anything	0.00%	0
Q11. Which one component of supracondylar fracture patient care is least likely to change in your hands, regardless of potential savings in time and cost?		
Settings in the OR (positioning, C-arm, draping, antibiotics, and anesthesia method)	14.29%	1
Fracture reduction and fixation technique	57.14%	4
Casting techniques and dressing materials (Xeroform, 4 × 4, color/white fiberglass/plasters…)	0.00%	0
Pain control (NSAIDs and narcotics)	0.00%	0
Peri-OP logistics (pre-OP admission vs. day surgery, etc., pre-OP splint, post-OP follow-up visit frequency, and management…)	0.00%	0
I am open to change in anything for cost and/or time savings	28.57%	2
Q12. If you changed to a standard operative technique for supracondylar humerus fracture, how much extra time would your first case require?		
0 minute	42.86%	3
∼5 minutes	14.29%	1
∼10 minutes	28.57%	2
∼15 minutes or more	14.29%	1

(B) Answer Choices	Responses (%, n)	
Q1. In your training up until now, how many operative supracondylar humerus fractures have you logged?		
10 cases or more	36.36%	8
∼6-9 cases	22.73%	5
∼3-5 cases	22.73%	5
∼1-2 cases	18.18%	4
Q2. How many cases did it take for you to be comfortable fixing a supracondylar humerus fracture in the OR independently?		
10 cases or more	4.55%	1
∼6-9 cases	13.64%	3
∼3-5 cases	18.18%	4
∼1-2 cases	4.55%	1
I still need more cases to be competent	59.09%	13
Q3. How many cases would it have taken you to be comfortable fixing a supracondylar humerus fracture in the OR independently, if every attending did it the same way? (patient setup, c-arm position, draping, reduction and pinning technique, casting, and dressing technique)		
10 cases or more	22.73%	5
∼6-9 cases	45.45%	10
∼3-5 cases	18.18%	4
∼1-2 cases	13.64%	3
Q4. At this point in your training, how much time would it take you, if you were to do a closed reduction and pinning for a supracondylar humerus fracture as the primary surgeon? (starting from the patient being anesthesitized to finishing the cast)		
∼15 minutes	4.55%	1
∼30 minutes	31.82%	7
∼60 minutes	36.36%	8
∼90 minutes or more	4.55%	1
I don't think I can do it as the primary surgeon	22.73%	5
Q5. Do you think it would benefit your training if every attending treated supracondylar humerus fractures operatively the same way?		
Yes	95.45%	21
No	4.55%	1
Q6. Please indicate your current level of training.		
PGY 1	18.18%	4
PGY 2	9.09%	2
PGY 3	36.36%	8
PGY 4	22.73%	5
PGY 5	9.09%	2
Fellow	4.55%	1
